# Characterization and genetic analysis of the complete chloroplast genome of *Schisandra chinensis* (Magnoliaceae: Schisandra), an herbal medicine from China

**DOI:** 10.1080/23802359.2019.1637291

**Published:** 2019-07-12

**Authors:** Zhuo Tian

**Affiliations:** College of Information Technology, Jilin Agricultural University, Changchun, China

**Keywords:** *Schisandra chinensis*, chloroplast genome, herbal medicine, genomics, genetic analysis

## Abstract

*Schisandra chinensis* is a deciduous woody vine plant that has been used in traditional medicine for thousands of years in China. In this study, we researched the complete chloroplast genome of *S. chinensis.* The complete chloroplast genome length is 147,779 bp, containing a large single-copy region (LSC) of 97,352bp, a small single-copy region (SSC) of 20,313 bp, and a pair of inverted repeat (IR) regions of 15,057 bp. The overall nucleotide composition is: A of 29.8%, T of 30.8%, C of 20.2%, and G of 19.2%, with a total GC-content of the chloroplast genome 39.4% and AT of 60.6%. Whole chloroplast genome of *S. chinensis* contains 126 genes, including 83 protein-coding genes (PCG), 35 transfer RNA (tRNAs) genes, and 8 ribosome RNA (rRNAs) genes. Phylogenetic and genetic analysis based on 30 herbal species confirmed the position of *S. chinensis* closely related to *S. sphenanthera*. The complete chloroplast genome of *S. chinensis* provides more molecular data for the genetic diversity and genetic evolutionary relationship of this species in China.

*Schisandra chinensis* is called Wu-wei-zi in China and has been used as traditional medicine in China for thousands of years (Lu and Chen 2009). It is a deciduous woody vine plant and belongs to the Schisandra family, which s is a horticultural plant with edible fruit and also used medicinally. It is distributed in Jilin, Heilongjiang, Liaoning, and Neimenggu provinces in China, as well as in Russia, Korea, Japan, and other Southeast Asian countries (Liu et al. 1996). It contains Schisandrin, Vitamin C, Resin, Tannin, and a small amount of sugar constituents in *S. chinensis*, which has the effects of astringent essence nourishing, relieving diarrhea, and perspiration (Zhang et al. 2018). At present, the research of *S. chinensis* is focused on its constituents. *Schisandra chinensis* genome has not been sequenced and analyzed yet, so these influence factors have limited the development of its genome research. In this study, we researched the complete chloroplast genome of *S. chinensis* and discussed the phylogenetic and genetic relationship of this herbal species, which provides more molecular data for the genetic diversity and contributes to the study of this species in future.

The whole plant specimen sample of *S. chinensis* was purchased from Wanliang town herbal medicine market (Wanliang, Jilin, China, 127.30E; 42.44N). The total genomic DNA was extracted from ripe fruit and stored in the Jilin Agricultural University Information Laboratory (No.JAUIL001), using Plant Tissues Genomic DNA Extraction Kit (TIANGEN, BJ, CN). The genomic DNA was sequenced based on the Illumina pair-end technology. The chloroplast (cp) genome was assembled and annotated using the MitoZ (Meng et al. 2019). The physical map of the chloroplast genome of *S. chinensis* was generated using OGDRAW (Lohse et al. 2013).

The accurate annotated complete chloroplast genome was submitted to GenBank with the accession number MK7146641. The complete chloroplast genome of *S. chinensis* was a circle with 147,779 base pairs (bp) in length, containing a large single-copy (LSC) region of 97,352 bp, a small single-copy (SSC) region of 20,313 bp, and a pair of inverted repeat (IRA and IRB) regions of 15,057 bp. The CP genome of *S. chinensis* comprised 126 genes, including 83 protein-coding genes (PCG), 35 transfer RNA genes (tRNAs), and 8 ribosomal RNA genes (rRNAs). In the IR regions, a total of 13 genes were found duplicated, including 4 PCG species (*ndhB, rps7, rps12 and ycf1*), 5 tRNA species (*trnV-GAC, trnI-GAU, trnA-UGC, trnR-ACG* and *trnN-GUU*), and 4 rRNA species (*rrn16, rrn23, rrn4.5* and *rrn5*). The base compositions of *S. chinensis* chloroplast genome is uneven and the overall nucleotide composition is: 29.8% A, 30.8% T, 20.2% C, and 19.2% G, with a total GC content of 39.4% and AT of 60.6%.

To understand the phylogenetic position of *S. chinensis* within the order of herbal plants, we selected and analyzed the chloroplast genomes of 29 herbal species with *S. chinensis* by phylogenetic and genetic analysis using the Maximum-Likelihood (ML) methods. ML analysis was performed using RAxML software (Stamatakis 2014) with the GTR + G + I model and used the number of bootstrap replicates with 5000. All of the nodes were inferred with strong support by the ML methods. The phylogenetic ML tree was represented using MEGA X (Kumar et al. 2018) and edited using iTOL Version 4 (Ivica and Peer 2019). The result (Figure 1) showed that the chloroplast genome of *S. chinensis* is clustered and the one closest to *S. sphenanthera* (No. NC_037145.1) provides more molecular data for the genetic diversity and genetic evolutionary relationship of herbal medicine in China.

**Figure 1.**
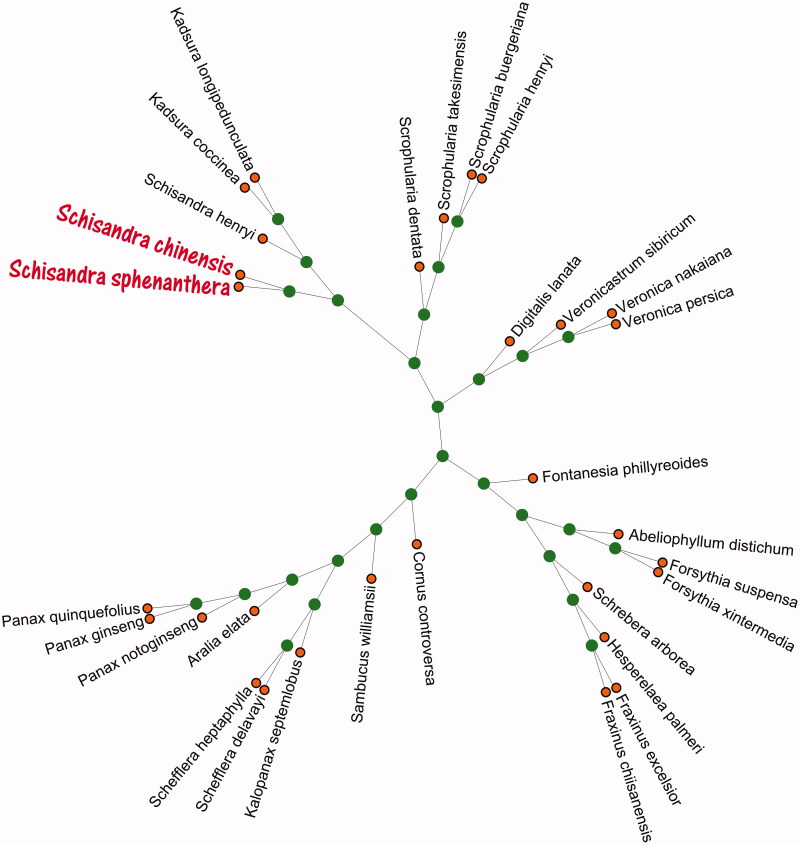
The phylogenetic and genetic relationships of 29 herbal species and *S. chinensis* based on chloroplast genome sequences. Numbers above each node indicates the ML bootstrap support values of 5000. The 29 herbal species accession numbers in this study have been deposited in the GenBank, Numbers are follows: *Abeliophyllum distichum* (KT274029.1), *Aralia elata* (KT153023.1), *Cornus controversa* (MG525004.1), *Digitalis lanata* (KY085895.1), *Fontanesia phillyreoides* (MG255754.1), *Forsythia suspensa* (MF579702.1), *Forsythia xintermedia* (MG255756.1), *Fraxinus chiisanensis* (MG214254.1), *Fraxinus excelsior* (MG594385.1), *Hesperelaea palmeri* (LN515489.1), *Kadsura coccinea* (MH029822.1), *Kadsura longipedunculata* (MH535482.1), *Kalopanax septemlobus* (KC456167.1), *Panax ginseng* (KF431956.1), *Panax notoginseng* (KP036468.1), *Panax quinquefolius* (KT028714.1), *Sambucus williamsii* (KX510276.1), *Schefflera delavayi* (KC456166.1), *Schefflera heptaphylla* (KT748629.1), *Schisandra henryi* (MH394370.1), *Schisandra sphenanthera* (NC_037145.1), *Schrebera arborea* (MG255767.1), *Scrophularia buergeriana* (KP718626.1), *Scrophularia dentata* (KT428154.1), *Scrophularia henryi* (MF861203.1), *Scrophularia takesimensis* (KM590983.1), *Veronica nakaiana* (KT633216.1), *Veronica persica* (KT724052.1), *Veronicastrum sibiricum* (KT724053.1).
